# Characterization of two Austrian porcine reproductive and respiratory syndrome virus (PRRSV) field isolates reveals relationship to East Asian strains

**DOI:** 10.1186/s13567-015-0293-x

**Published:** 2016-01-11

**Authors:** Leonie J. Sinn, Leonie Zieglowski, Hanna Koinig, Benjamin Lamp, Bettina Jansko, Georg Mößlacher, Christiane Riedel, Isabel Hennig-Pauka, Till Rümenapf

**Affiliations:** Institute of Virology, Department for Pathobiology, University of Veterinary Medicine Vienna, Veterinärplatz 1, 1210 Vienna, Austria; Clinic for Swine, Department for Farm Animals and Veterinary Public Health, University of Veterinary Medicine Vienna, Veterinärplatz 1, 1210 Vienna, Austria; Animal Health Service Upper Austria, Molkereistraße 5, 4910 Ried im Innkreis, Austria

## Abstract

Porcine reproductive and respiratory syndrome virus (PRRSV) causes major problems for the swine industry worldwide. Due to Austria’s central location in Europe, a large number of animals are transported through the country. However, little is known about current PRRSV strains and epidemiology. We determined full-length genome sequences of two Austrian field isolates (AUT13-883 and AUT14-440) from recent PRRSV outbreaks and of a related German isolate (GER09-613). Phylogenetic analysis revealed that the strains belong to European genotype 1 subtype 1 and form a cluster together with a South Korean strain. Remarkably, AUT14-440 infected the simian cell line MARC-145 without prior adaptation. In addition, this isolate showed exceptional deletions in nonstructural protein 2, in the overlapping region of glycoprotein 3 and 4 and in the 3′ untranslated region. Both Austrian isolates caused similar lung lesions but only pigs infected with AUT14-440 developed clear clinical signs of infection. Taken together, the genetic and biological characterization of two novel Austrian PRRSV field isolates revealed similarities to East Asian strains. This stresses the necessity for a more detailed analysis of current PRRSV strains in Europe beyond the determination of short ORF5 and ORF7 sequences.

**Electronic supplementary material:**

The online version of this article (doi:10.1186/s13567-015-0293-x) contains supplementary material, which is available to authorized users.

## Introduction

Porcine reproductive and respiratory syndrome virus (PRRSV) is one of the most economically important pathogens in the swine industry worldwide [1, 2]. It is the etiological agent of porcine reproductive and respiratory syndrome (PRRS), which is characterized by respiratory disorders as well as by growth retardation in growing pigs and reproductive failure in late gestation sows [3, 4]. The disease emerged in the late eighties in North America, with the first outbreaks in Europe recorded in 1990 [3].

The causative agent, PRRSV, was first isolated in 1991 in the Netherlands [4]. This strain, Lelystad virus (LV), is regarded as the prototype strain of European PRRSV type 1 (PRRSV-1), whereas VR2332 represents the North American PRRSV type 2 (PRRSV-2). The two genotypes share only about 60% identity at the nucleotide level [5]. Due to high mutation and recombination rates [6], variability is also high within the genotypes, especially in type 1. Therefore, three subtypes have been proposed for PRRSV-1 based the size of the nucleocapsid protein (N): pan-European subtype 1 and Eastern European subtypes 2 and 3 [7].

PRRSV is a small, enveloped virus with a single-stranded positive-sense RNA genome that belongs to the family *Arteriviridae*, order *Nidovirales* [8]. The 5′-capped and 3′-polyadenylated genome of PRRSV is about 15 kb in length and contains ten open reading frames (ORF) [9, 10]. ORF1a and 1b constitute over 75% of the viral genome and encode two polyproteins, which are cleaved into at least 14 nonstructural proteins (nsp) that are responsible for genome replication and transcription [11]. ORF2–4 encode the minor structural proteins, including glycoprotein (GP) 2, 3 and 4, which form a hetero-trimer that is believed to be involved in virus entry [12]. The major structural proteins GP5, membrane protein (M) and N protein are encoded by ORF5–7. Nucleotide sequences of ORF5 (603–606 bp) and ORF7 (372–387 bp) are widely used for phylogenetic studies. However, the analysis is limited as these short genomic sequences might be subject to recombination and immunological selection pressure [6]. Phylogenetic analysis based on ORF5 and ORF7 may also lead to different subtyping of PRRSV-1 strains [13]. For these reasons, the use of further methodologies for genetic subtyping has been suggested, such as whole genome sequencing [6, 14]. The lack of published full-length sequences is exemplified by the situation in Austria, where the limited data on current strains consists solely of ORF5 or ORF7 sequences [15–17]. This gap in knowledge complicates the identification of the source of outbreaks, which is crucial as the most important PRRSV problems in Austria occur as a consequence of (re-) introduction into formerly free herds. An awareness of recent isolates and genomic changes that possibly lead to lower protection is also important in terms of assessing the effectiveness of vaccines.

The aim of this study was to determine the etiological agents of two PRRSV-1 outbreaks in Austria and to characterize them genetically and biologically. The results reveal that the isolated PRRSV-1 subtype 1 field strains (AUT13-883 and AUT14-440) have genetic and phylogenetic similarities to East Asian strains.

## Materials and methods

### Viruses and cells

Wild-type PRRSV strains AUT13-883 and AUT14-440 were isolated from sera of two farms in Upper Austria in 2013 and 2014. A cell culture adapted PRRSV-1 subtype 1 strain (GER09-613) that was isolated from the field in Germany in 2009 was also included in the analysis (kindly provided by L. Haas, TiHo Hannover). MARC-145 cells (CCLV RIE 277 [18]) were obtained from the Friedrich-Loeffler-Institute in Germany. Preparation of porcine alveolar macrophages (PAM) was essentially performed as described previously [19]. Briefly, lungs of 6- to 12 week-old PRRSV-free, healthy pigs were washed 2–3 times with PBS (pH 7.2). Aliquots of lavage fluid were pooled and centrifuged for 5 min at 1000 *g*. The cells were washed in PBS and repelleted twice. For storage, PAM were resuspended in FCS supplemented with 10% dimethyl sulfoxide and frozen at −150 °C. MARC-145 cells were cultured in Dulbeccos’s modified Eagle’s medium (Life Technologies, Waltham, USA) supplemented with 10% fetal calf serum (Bio&Sell, Nürnberg, Germany) and antibiotics (100 U/mL penicillin and 100 µg/mL streptomycin). Two additional antibiotics (enrofloxacin and kanamycin) were added for the culture of PAM. All cells were maintained at 37 °C and 5% CO_2_ and observed daily for cytopathic effects.

### Sample collection

Serum samples were collected from two medium-sized piglet producers in Upper Austria in November 2013 (farm 883) and March 2014 (farm 440). Both farms were experiencing PRRSV-related problems such as pneumonia, wasting, variance in growth, conjunctivitis and coughing in rearing piglets, as well as reproductive disorders such as mummification and abortions in sows. Whereas one farm (883) had experienced minor problems related to PRRSV over a period of 2 years, the other (440) was formerly free of PRRSV and had losses of up to 50% in one farrowing badge. Serum samples from both farms had been tested positive for PRRSV by qRT-PCR by the Animal Health Service Upper Austria. Neither of the farms had used vaccines against PRRSV.

### Virus isolation and titration

For virus isolation, 50 µL serum of diseased piglets from farms 440 and 883 were used to inoculate 5 × 10^6^ PAM on a six-well format. After 2 days, the cell culture supernatant was clarified by centrifugation (5 min, 3000 *g*) and passaged on PAM and MARC-145 cells. Virus infection was generally analysed 2 days post-infection by immunofluorescence staining. Virus titres of supernatants were confirmed on PAM from a single animal to ensure comparability and expressed as tissue culture infectious dose of 50% (TCID_50_). In the animal trial a viral stock from the 6^th^ to 8^th^ passage was used for inoculation.

### Detection of PRRSV-positive cells

Cells were fixed with 1:2 methanol-acetone for 2 min at room temperature and air-dried. Anti-PRRSV-N-protein monoclonal antibodies (clone P10/b1) [20] (kindly provided by A. Saalmüller, Vienna) were employed for the detection of infected cells. Goat anti-mouse conjugated with Cy3 (Dianova/Jackson, Hamburg, Germany) was used as a secondary antibody.

### Determination and analysis of full-length genome sequences

Total RNA was extracted from PAM 5 days after inoculation and from serum samples using the RNeasy Mini Kit and the QIAamp Viral RNA Mini Kit (Qiagen, Hilden, Germany) according to the manufacturer’s instructions. RNA was eluted in 30 µL distilled water and either used immediately for RT-PCR or stored at −80 °C for subsequent analysis.

RT-PCR was carried out using the LongAmpKit (NEB, Ipswich, USA) or the One Step RT-PCR Kit (Qiagen, Hilden, Germany) according to the manufacturer’s instructions. Seven primer pairs that were highly conserved in PRRSV-1 strains were designed (available upon request) based on published sequences available in NCBI GenBank. The resulting PCR amplicons were overlapping and covered the whole genome.

The DNA fragments were subjected to gel electrophoresis, purified by the peqGOLD Gel Extraction Kit (Peqlab, Erlangen, Germany) and sequenced by commercial laboratories (Microsynth Austria, Vienna, Austria and Eurofins Genomics, Ebersberg, Germany). Primer selection for sequencing was based on available PRRSV-1 sequences. The determined full-length genome sequences of GER09-613, AUT14-440 and AUT13-883 were submitted to GenBank (KT344816, KT334375 and KT326148).

Initial phylogenetic analysis was carried out with NCBI’s Basic Local Alignment Search Tool for nucleotides (BLASTn). The two closest neighbours of the isolates presented in this study were determined for ORF5 and ORF7. Pairwise comparison and identity calculations were carried out with CLC Main Workbench 7.6 (CLCBIO, Aarhus, Denmark). Alignments and phylogenetic trees were generated with the software CLC Sequence Viewer 7.6 (CLCBIO, Aarhus, Denmark) with bootstrap values based on 1000 replicates. All PRRSV-1 strains with full genome sequences deposited in GenBank were used to construct the phylogenetic trees. For the trees based on ORF5 and ORF7, the two closest neighbours—as determined by NCBI BLASTn—were added. The PRRSV-2 prototype VR-2332 was used as an out-group. Recombination analysis was performed with the recombination analysis tool (RAT) [21] using the full genome alignment of PRRSV-1 strains.

### Animal trial

#### Animals

Twenty-seven-week-old conventional pigs (#1–20) were obtained from a PRRSV-negative herd and housed in a biosafety level 2 facility. They had been vaccinated against PCV-2 and *M. hyopneumoniae* in their third week of life. To confirm their PRRSV status nasal swabs taken at the time of arrival were analysed by qRT-PCR and serum samples were tested for antibodies against PRRSV with the commercial IDEXX X3 ELISA (IDEXX laboratories, Westbrook, USA). The animal experiments were approved by the ethics committee of the University of Veterinary Medicine, Vienna and the Federal Ministry of Science, Research and Economy (BMWF-68.205/0196-WF/V/3b/2014).

#### Serum titration

Tenfold dilution series of sera were prepared and transferred to naïve PAM. For each time point and each animal, two replicates were performed. Three days later cells were fixed and stained and the TCID_50_ was calculated.

#### Quantitative reverse transcription-PCR (qRT-PCR)

To detect viremia, viral RNA was extracted from 140 µL serum with QIAamp Viral RNA Kit (Qiagen, Hilden, Germany) according to the manufacturer’s instructions. RNA was eluted in 60 µL distilled water and 1 µL was directly used for amplification with the KAPA™ SYBR^®^ FAST One-Step qRT-PCR Kit (Peqlab, Erlangen, Germany) on an ABI 7500 cycler (Applied Biosystems, Foster City, USA). Published primers Pesch PLS (5′-ATGGCCAGCCAGTCAATC-3′) and Pesch PLR (5′-TCGCCCTAATTGAATAGGTG-3′) were used for amplification [22]. A recombinant cDNA clone of a PRRSV-1 strain was purified using the QIAGEN Plasmid Midi Kit (Qiagen, Hilden, Germany) and spectrophotometrically quantified. The copy number of recombinant plasmids was calculated with the formula: N (molecules per µL) = [C (DNA concentration in µg/µL)/K (fragment size in bp)] × 185.5 × 10^13^ (factors derived from DNA weight, volume and the Avogadro constant). To obtain a standard curve, a tenfold dilution series of cDNA of the PRRSV-1 clone was included in the qRT-PCR setup. Cycling conditions were 42 °C 5:00, 95 °C 5:00 and 42 cycles of 95 °C 0:03, 60 °C 1:00 and 80 °C 0:33 (fluorescence detection step) followed by a dissociation step (95 °C 0:15, 60 °C 1:00, 95 °C 0:15). The numbers of genome copies were calculated with the 7500 System SDS Software (Applied Biosystems, Foster City, USA) based on the standard curve and projected to 1 mL serum by multiplication by 428.4.

To detect viral shedding, nasal swabs were submerged in 2 mL of sterile saline (0.7% NaCl) and thoroughly shaken for 30 s. 200 µL of supernatant were used for viral RNA extraction with the High Pure Viral RNA Kit (Roche Diagnostics, Vienna, Austria) according to the manufacturer’s instructions. RNA was eluted in 50 µL of the kit’s elution buffer and 13.6 µL were directly used for amplification with the LightCycler^®^ RNA Amplification Kit SYBR Green I with a LightCycler^®^ 480-II System (Roche Diagnostics, Vienna, Austria). The nested RT-PCR from Pesch [21] was adapted for detection with SYBR Green I^®^. Reverse transcription and activation was performed according to the manufacturer’s instructions, followed by 32 cycles of 60 °C 0:10, 72 °C 0:22 and 95 °C 0:10. The nested amplification step was carried out with LightCycler^®^ FastStart DNA Master(plus) SYBR Green I (Roche Diagnostics, Vienna, Austria) using 2 µL of the first PCR reaction. Cycling conditions were 25 cycles of 60 °C 0:10, 72 °C 0:20 and 95 °C 0:20. The fluorescence detection step was carried out at 84 °C. The PCR was followed by a dissociation step. Results were considered positive when a clear amplification curve was observed and the melting temperature of the amplicons (calculated by LightCycler^®^ 480 System Software) was within the expected range.

#### Serology

Serum samples were tested for antibodies against PRRSV with the commercial IDEXX X3 ELISA (IDEXX laboratories, Westbrook, USA) according to the manufacturer’s instructions. Ratios of optical densities of sample and positive control (P/PK ratios) were calculated and considered positive when >0.4.

### Statistical analysis

Data were analysed with the SPSS statistics software (SPSS Ltd, Quarry Bay, Hong Kong). Oneway ANOVA with Posthoc LSD test was applied for the analysis of all results except for those from the ELISA, which were analysed with the t test with the test value 0. A *p* value < 0.05 was considered statistically significant.

## Results

### Virus isolation

Two PRRSV-1 outbreaks in 2013 (farm 883) and 2014 (farm 440) were brought to our attention by the responsible veterinarians. Case 883 was characterized by mild respiratory disorders and a low virus load that persisted over 2 years. In case 440, suckling piglet mortality reached 50%, which is severe for PRRSV outbreaks in Austria, and abortions were observed. Both farms had been found to be PRRSV-1 positive by qRT-PCR before we obtained any samples. To investigate the PRRSV strains responsible for the outbreaks, we isolated two viruses from the serum of diseased piglets after serial inoculation of PAM (termed AUT13-883 and AUT14-440). MARC-145 cells were also susceptible for AUT14-440 without adaption but were not infected by AUT13-883.

### Phylogenetic analysis

The sequences of the ORF5 and 7 genes revealed that both isolates belong to PRRSV-1 subtype 1 [7] but the results for ORF5 and ORF7 were not consistent. In the phylogenetic tree based on ORF5, AUT14-440 clustered together with German and South Korean PRRSV-1 isolates (among them KNU-07) (Additional file 1). AUT13-883 represented a different branch of the tree and was most closely related to German strain EU-2a from 1992 [23] and to Austrian strain 2888 [15]. In contrast, when ORF7 sequences were used for phylogenetic analysis, the two Austrian isolates formed a group together with Korean strain KNU-07 [24], a Croatian strain [25] and German strains [26] (Additional file 2).

**Figure 1:**
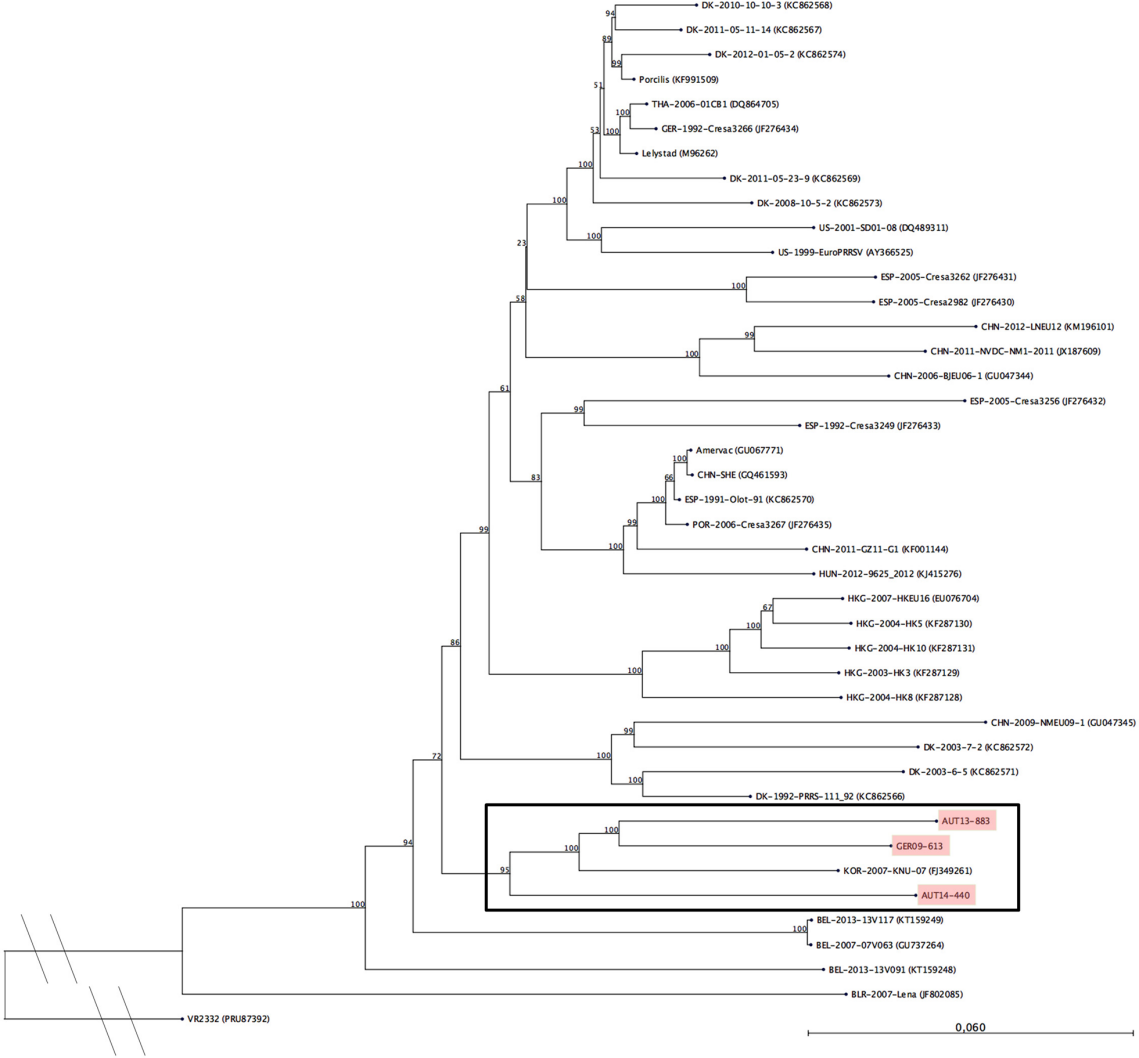
**Phylogenetic analysis based on nucleotide sequences of full-length genomes of 41 PRRSV-1 strains and PRRSV-2 prototype VR2332 as an out-group.** The PRRSV strains presented in this study are marked in red and the associated sub-tree is highlighted with a box. The tree was constructed using the neighbour joining method with the numbers at the nodes representing bootstrap values in % of 1000 replicates. Scale bar: number of substitutions per site.

Since the results of the phylogenetic trees based on ORF5 and ORF7 were inconclusive, complete genome sequences for AUT13-883 and AUT14-440 were determined (GenBank: KT326148 and KT334375). A phylogenetic tree based on the full genome sequences of the isolates presented in this study, 38 published PRRSV-1 strains and PRRSV-2 prototype VR-2332 (Figure 1) confirmed the relationship between the two Austrian isolates, GER09-613 and KNU-07. All four strains cluster together, with no other strain in the same branch. AUT13-883 and GER09-613 are the most closely related strains.

### Distinct deletions in the genome of AUT14-440

**Figure 2:**
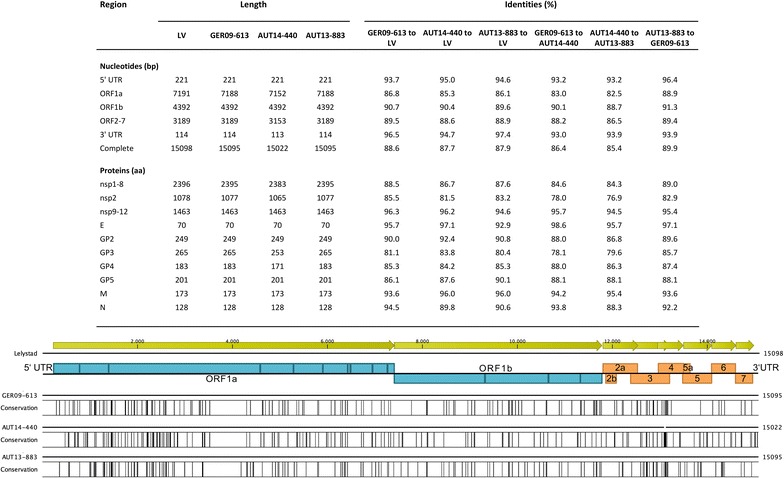
**Detailed comparison of complete genomes of GER09-613, AUT14-440, AUT13-883 and Lelystad virus (LV) (table) and schematic overview of nucleotide differences between LV and the isolates presented in this study.** Black bars represent areas with a low degree of similarity to LV. Open reading frames (ORF) of PRRSV that encode non-structural proteins (blue) and structural proteins (orange) are shown.

**Figure 3:**
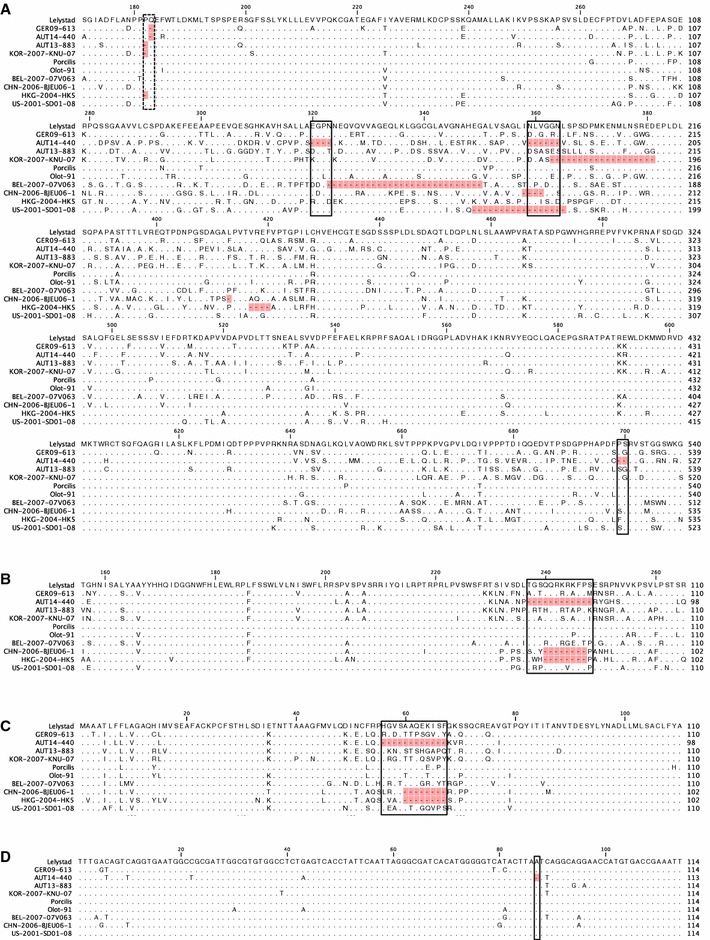
**Multiple alignments of partial nsp2** (**A**)**, GP3** (**B**) **and GP4** (**C**) **amino-acid sequences of eleven different European PRRSV strains.** Only aa differing from Lelystad virus (LV) are shown, with identical aa represented by dots. Deletions compared to LV are marked in red. Deletions of AUT14-440 are highlighted with solid boxes; a deletion in all three isolates is indicated with a dotted box. The numbers above the alignment indicate the position in the protein. **A** A single aa deletion is present in all three isolates and KNU-07 at position 182 or 183 in nsp2. In AUT14-440 aa 320–323, aa 359–364 and aa 699–700 are deleted compared to LV. **B, C** The box marks a 12 aa deletion in the overlapping region of GP3 and GP4 of AUT14-440. **D** Multiple alignment of 3′-UTR nucleotide sequences of ten European PRRSV strains. Nucleotides differing from Lelystad virus (LV) are shown, with identical nucleotides represented by dots. A deletion of 1 nt at position 87 in AUT14-440 is marked in red and highlighted with a solid box.

Assembly of the overlapping sequences resulted in complete genomes consisting of 15022 nucleotides (nt) for AUT14-440 and 15095 nt for GER09-613 and AUT13-883, excluding the 3′ poly(A) tails. Detailed comparison to the European prototype LV revealed marked differences in the region of nsp2. Both Austrian virus isolates and the German strain GER09-613 possess a shorter ORF1a due to deletions in nsp2 (Figure 2). The difference of three nucleotides in ORF1a between AUT13-883 (7188 nt) and LV (7191 nt) results in the deletion of proline 182 in nsp2 (Figure 3A, dotted line box). The deletion is also present in the South Korean strain KNU-07 and has been described for strains from Hong Kong. GER09-613 and AUT14-440 (ORF1a lengths of 7188 and 7152 nt) share a deletion of three nt, resulting in the loss of glutamine 183 in nsp2, one amino acid (aa) downstream of proline 182 (Figure 3A, dotted line box). In comparison to LV, AUT14-440 has further deletions in nsp2 at aa positions 320–323, aa 359–364 and aa 699–700, all in hypervariable regions of nsp2 where deletions are frequently found (Figure 3A, solid line boxes). ORF3 and ORF4 of AUT14-440 were also truncated by 36 nt when compared to LV, resulting in a deletion of 12 aa in the overlapping region of GP3 and GP4 (Figures 3B and C solid line boxes). Shorter deletions (up to 8 aa) in this area have been described for several strains, including HK5 and the Chinese isolate BJEU06-1. AUT14-440 also shows a single nucleotide deletion at position 87 in the 3′ untranslated region (UTR) (Figure 3D, solid line box). All deletions in virus isolate AUT14-440 were also found when RNA from the original serum sample was used for RT-PCR and PCR products were directly sequenced.

Pairwise nucleotide comparison of the complete genome resulted in identities of 87.7, 87.9 and 88.6% with LV for AUT14-440, AUT13-883 and GER09-613. Identities between the isolates were 86.4% (GER09-613 to AUT14-440), 85.4% (AUT14-440 to AUT13-883) and 89.9% (AUT13-883 to GER09-613) (Figure 2).

### Determination of virulence

Genetic and phylogenetic analysis showed a striking relationship between the Austrian isolates, the German strain GER09-613 and the South Korean strain KNU-07. The reported severity of the clinical symptoms associated with AUT13-883 and AUT14-440 differed markedly, while the clinical symptoms of GER09-613 have not been described. However, the particular clinical signs in an affected herd provide no more than an indication of the pathogenicity of a PRRSV isolate, as other pathogens may be involved. To assess the virulence and to test Koch’s postulates, an animal trial was performed with the Austrian isolates AUT13-883 and AUT14-440 and the German isolate GER09-613, which clusters in the same branch of the phylogenetic tree.

**Figure 4:**
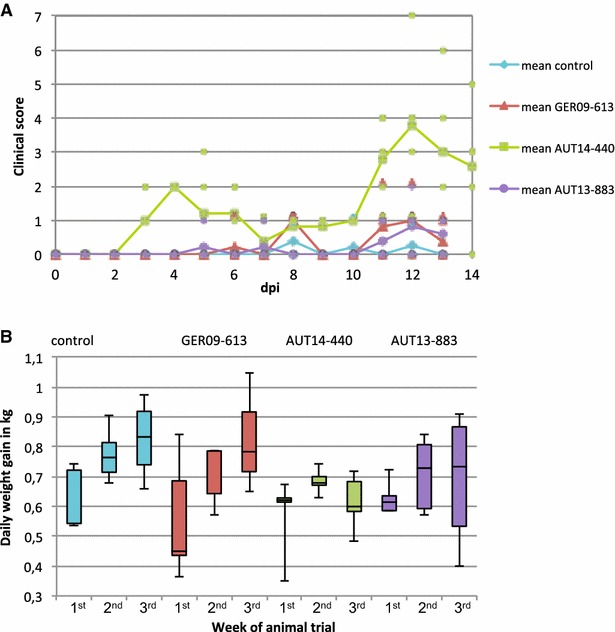
**Clinical signs and daily weight gain. A** Pigs were examined daily and a clinical score was calculated based on the severity of PRRSV-associated clinical signs. The mean values for each group are shown with a continuous line and single spots represent individual animals. As group AUT14-440 was euthanized on day 14 pi there are values for 1 day more than for the other groups. **B** Pigs were weighed at six time points and daily weight gain was calculated for three time frames: 1st week (−7 to 0 dpi), 2nd week (0 to 7 dpi) and 3rd week (7 to 13 dpi) of the trial. Whiskers represent maximum and minimum values. Differences between the groups were visible for the last time period, 7–13 dpi, although they are not statistically significant (*p* = 0.055 between GER09-613 and AUT14-440).

PRRSV-seronegative pigs were distributed equally into four groups according to their weight and sex. Each group of five animals was housed in a separate compartment to avoid cross-contamination. After 1 week of adaptation (day 7 to day 0), pigs from groups GER09-613, AUT13-883 and AUT14-440 were inoculated intranasally with 3 mL medium (1.5 mL in each nostril) containing 1 × 10^5^ TCID_50_ of the respective strain. Pigs from the control group received the same amount of medium without virus. Clinical signs and rectal temperature were monitored daily. A scoring system was applied that rates liveliness, dyspnoea, coughing, nasal and ocular discharge, conjunctivitis and cyanosis with scores from 0 (physiological) to 3 (severe clinical signs). Daily scores for each animal were added to obtain a value for the overall health status and the mean clinical scores for the four groups were calculated (Figure 4A). All pigs infected with AUT14-440 showed mild to moderate dyspnoea on three or more study days. Other clinical signs that occurred frequently in all animals in this group were apathy and conjunctivitis. Two waves of clinical signs could be distinguished: the first wave occurred around day 4 post infection (pi) and the second, with more prominent signs of illness, took place around day 12 pi. In the other infection groups, only one (AUT13-883) or two (GER09-613) animals showed mild dyspnoea for no more than 2 days in a row. Pigs in the control group showed no relevant clinical signs in the trial, except for animal #4 that had to be euthanized on study day 10 due to a severe lameness in the hind legs. There was no significant difference in rectal temperature between the four groups.

The pigs were weighed at −7, 0, 3, 7, 10 and 13 days post infection (dpi). For each week of the trial (study days −7 to 0, 0 to 7, 7 to 13) daily weight gain was calculated and is shown for each group as a box plot with maximum and minimum values represented as whiskers (Figure 4B). From the first week to the second week of the trial, all groups showed an average increase of daily weight gain between 0.08 and 0.16 kg. From the second to the third week, the control group and group GER09-613 showed a mean increase of daily weight gain of 0.05 and 0.11 kg, whereas in the groups infected with the Austrian field isolates daily weight gain did not increase (mean values of −0.07 kg for AUT14-440 and −0.02 kg for AUT13-883). Despite the apparent differences between the groups, the figures are not statistically significant due to the small group size and the high variation within the groups (*p* = 0.055 between GER09-613 and AUT14-440 for the third week).

### Gross pathology and histopathology

**Figure 5:**
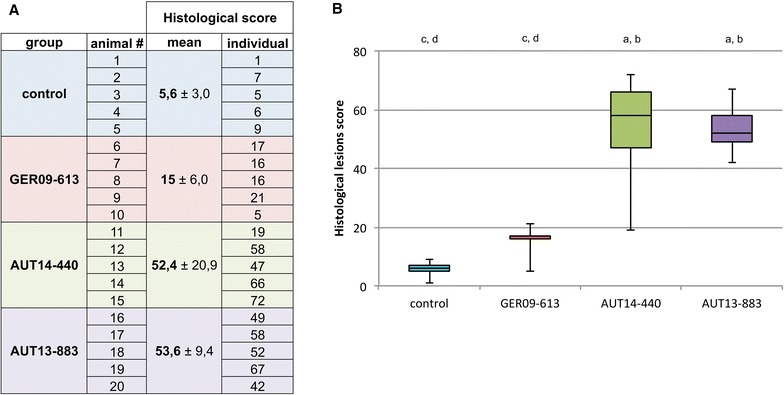
**Histological lung lesions.** For each animal, a histopathological lung lesion score was calculated by considering the degree of PRRSV-associated lesions in every lung lobe. Histological lesion scores are presented **A** in a table and **B** as a box-plot. Mean score (±standard deviation) and individual scores for each animal are displayed for all groups. **B** Whiskers represent maximum and minimum values. Letters indicate significant differences (*p* < 0.01) from control group (a), group GER09-613 (b), group AUT14-440 (c) and group AUT13-883 (d).

At 13 and 14 dpi, pigs were euthanized and necropsied with special emphasis on the lungs. No other organs than lungs showed pathological lesions. Samples from each lung lobe were fixed in formalin, embedded in paraffin, stained with hematoxylin and eosin and examined histologically. Lesions were hypertrophy and hyperplasia of pneumocytes, septal infiltration by mononuclear cells, perivascular and intraalveolar accumulation of inflammatory cells and necrotic debris. Depending on their occurrence in different lung lobes and on the severity of the lesions, a total score per lung was calculated, applying a PRRSV specific scoring system that has been described elsewhere [27]. The mean values for the Austrian isolates were 52.4 ± 20.9 for AUT14-440 and 53.6 ± 9.4 for AUT13-883 (Figure 5A). In these two groups all types of lesions mentioned above were present in mild to severe manifestations with no visible differences between the groups. The results differed significantly (*p* < 0.01) from both the control group with a mean score of 5.6 ± 3.0 and group GER09-613, which mainly showed minor accumulations of inflammatory cells (mean histological score of 15 ± 6.0). The scores for each group are displayed as a boxplot in Figure 5B.

### Viremia and virus shedding

To exclude the possibility of cross-contamination between the groups, 50 µL of serum taken 3 dpi from one animal per group (#10, 14, 19) were inoculated onto naïve PAM and virus was re-isolated. RT-PCR and subsequent sequencing of the 700 bp PCR amplicon covering ORF4 confirmed the identity of the viral strains. Compared to the parental strain, two point mutations were detected in each strain. One of them was the same for all three strains and resulted in an amino-acid change from aspartic acid to asparagine at position 33 in GP4. In addition melting temperatures of amplicons from positive nasal swabs confirmed that no cross-contamination had occurred (data not shown).

**Figure 6:**
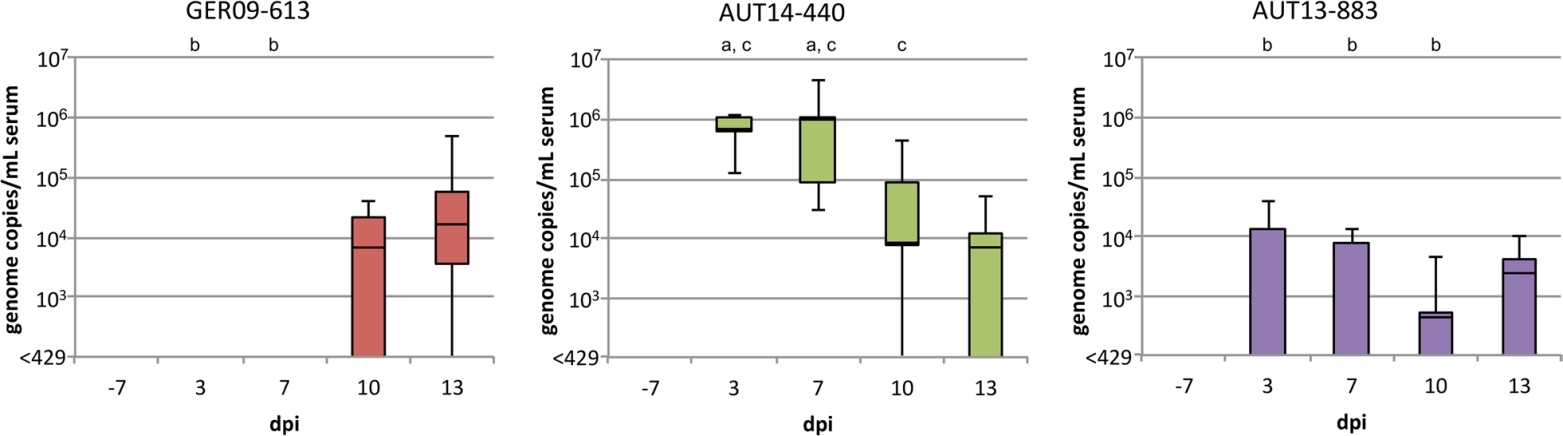
**Viral load in serum quantified by qRT-PCR.** For each infected group qRT-PCR results are displayed as genome copies per mL serum for −7, 3, 7, 10 and 13 dpi. Whiskers represent maximum and minimum values. Letters indicate significant differences (*p* < 0.05) to group GER09-613 (a), AUT14-440 (b) and AUT13-883 (c).

Viral nucleic acids in serum could be detected with qRT-PCR as early as day 3 pi in all animals from group AUT14-440 and in two animals from group AUT13-883 (Figure 6). In the GER09-613 group, the first virus-positive animals were detected 10 dpi. On day 13 pi all animals from this group except one (#8) tested positive for viral RNA; animal #8 remained negative for qRT-PCR as well as for titration on PAM (data not shown) on all sampling days. The number of genomic copies in the serum of animals infected with AUT14-440 declined towards the end of the trial. In the AUT13-883 group, the onset of viremia differed between the pigs. The maximum number of genome copies per mL serum was up to 100 times higher in the AUT14-440 group than in the other groups. On days 3 and 7 pi, the difference between group AUT14-440 and all other groups was significant (*p* < 0.01), whereas on day 10 pi only the difference to the control group and the AUT13-883 group was significant (*p* < 0.05). Serum titres on PAM were also highest in the AUT14-440 group (up to 2.3 × 10^3^ TCID_50_/mL, data not shown). Viral RNA or infectious virus could be detected in the serum of all infected animals, except for #8, with qRT-PCR and titration on PAM on one or more sampling days. The animals from the control group gave negative results with both assays throughout the trial.

To examine virus shedding from the upper respiratory tract, nasal swabs were taken at −7, 3, 7, 10 and 13 dpi and analysed with qRT-PCR for PRRSV-specific nucleic acids (data not shown). All animals from infected groups were virus-positive 3 dpi. Group GER09-613 tested completely negative on day 7 pi and one pig (#8) remained negative until the end of the trial, while the other animals tested positive on day 10 and 13 pi. Virus shedding could be determined for four pigs each from groups AUT14-440 and AUT13-883 on study days 7 and 10, whereas only two animals from group AUT14-440 and all animals from group AUT13-883 were positive on the last sampling day, day 13. The nasal swabs from the control group tested negative for PRRSV-specific nucleic acids at every time point.

### Serology

**Figure 7:**
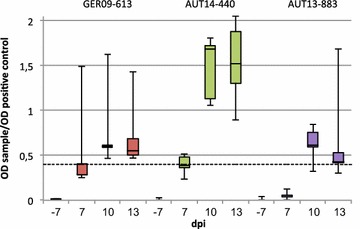
**Antibody response to PRRSV infection.** ELISA IDEXX X3 results as a ratio of the optical density (OD) of the sample and the OD of the positive control for infected groups at −7, 7, 10 and 13 dpi. Values over the cut-off at 0.4 (dotted line) were regarded as positive. Whiskers represent maximum and minimum values.

Antibody titres against PRRSV were determined at the beginning of the trial to ensure the PRRSV-free status of the animals. At this time point all pigs tested negative for N-specific antibodies with the commercial IDEXX X3 ELISA (Figure 7). Pigs were further screened for seroconversion 7, 10 and 13 dpi. First detection of PRRSV-specific antibodies was possible 7 dpi in the sera of two pigs from groups AUT14-440 and GER09-613. On days 10 and 13 pi all infected animals but one (#19) tested positive in ELISA. The clearest antibody response with the highest P/PK ratios was seen in the AUT14-440 group. The animals from the control group were negative for PRRSV-specific antibodies at every time point.

## Discussion

Austria is surrounded by seven neighbouring countries and lies on the border between Western and Eastern Europe. Due to its central location it has become a major European corridor for travel and the movement of goods, including live pigs. In fact, one of the isolates (AUT440-14) originated from a farm directly adjacent to the major west-east transit road. The reintroduction of PRRSV into virus-free herds is the most urgent yet least understood problem for the swine industry in Austria. Epidemiological evidence is often insufficient as only few ORF5 and ORF7 sequences from Austria are accessible at GenBank, most of them originating from only three publications [15–17]. In none of these cases were sequence data correlated to the biological properties of the isolates.

In this study, the initial question about the phylogenetic background of two field strains not only led to complete genome sequences of two recent Austrian field isolates but also to their characterization in vivo. (Phylo-) genetic information could be interpreted in the light of the phenotypic properties of the isolates. This is especially important because genetic relationships do not give evidence for the virulence of PRRSV strains [6]. To go beyond the descriptive determination of selected sequences, it is necessary to address the pathogen’s properties by carefully characterizing the isolate.

The initial virus isolation in this study was performed on primary PAM, which are believed to be the main target cells of PRRSV in vivo and are widely used for PRRSV-1 isolation and propagation [28]. The much more convenient simian cell line MARC-145 can usually not be used for unadapted PRRSV-1 virus strains, although it is readily infected by PRRSV-2 without adaptation [6, 28]. Interestingly, it also supported growth of the first passage of AUT14-440, a PRRSV-1 isolate. This observation encouraged us to investigate the phylogenetic classification of the strain.

Virus isolation is rarely performed in routine PRRSV diagnostics and sequences are normally obtained directly from clinical samples. Generally, only ORF5 or ORF7 sequences are determined. However, there are doubts about the validity of phylogenetic trees based solely on ORF5 or ORF7 sequences because of the recombination potential and high rate of nucleotide substitution shown by these genes [6, 14]. As a matter of fact, in the phylogenetic trees we determined based on ORF5 and 7 the relationship between the two Austrian isolates and closely related strains remained unclear as AUT13-883 grouped differently in the two trees. The difference might relate to recombination events, which are believed to have a major impact on PRRSV diversity [6, 29]. No evidence for this phenomenon was found by recombination analysis, probably due to the small number of full PRRSV-1 genomes available in GenBank (data not shown). This finding underlines the need for more full-length genome sequencing. The phylogenetic tree based on full genomes clearly showed relatedness between the two Austrian isolates, the German strain GER09-613 and the South Korean isolate KNU-07. However, it has to be considered that the four strains cluster together in a phylogenetic tree despite not being closely related (identities between 85.4 and 90.2%) and that only 38 PRRSV-1 strains were available for comparison, most of them from earlier than 2010. The picture might be less clear if the full genome sequences of more (recent) strains were available for inclusion in the analysis. At present we can only speculate whether the South Korean strain was imported to Europe or whether the cluster is the result of convergent evolution. It is also possible that all four strains originate from older strains from Germany (e.g., BH_95_10-08_EU) that show close relationships in ORF5 and ORF7 but for which complete sequences are not available. Detailed epidemiological statements will not be possible without a broader range of full-length PRRSV genomes.

Nevertheless, comparing not only a small part of the 15 kb PRRSV genome but full sequences revealed remarkable characteristics of the strains and provided indications that the four strains have a common origin. AUT13-883 and KNU-07 have a proline deletion in nsp2 that has only been noted in several strains from Hong Kong [30]. AUT14-440 and GER09-613 also share a single aa deletion in nsp2, a single position after the proline. Additionally, on either side of the 12 aa deletion in GP3 in AUT14-440 the four strains share areas of aa sequences that are very similar to one another but clearly differ from those of other PRRSV-1 strains, providing further indications of a possible relationship.

A closer look at the full genome sequences revealed additional deletions in strain AUT14-440. In nsp2, three more deletions of 2–6 aa support the conclusion from the phylogenetic analysis that the isolate is not as closely related as the three other strains. The 12 aa deletion in the overlapping region of GP3 and GP4 is unique to this strain. Other published isolates that carry a deletion in this highly variable area only miss 1–8 aa compared to LV and the concomitant occurrence of deletions in nsp2 and ORF3/4 has only been described for a few Chinese strains [31–33]. The first strains carrying a deletion in the overlapping region of ORF3 and ORF4 were isolated in Denmark as long ago as 1992, shortly after the discovery of PRRSV. In subsequent evaluations, deletion mutants were reported to evolve more quickly than non-deleted viruses [34]. This might be due to immunological pressure, since neutralizing antibodies against this region in GP4 have been described in vitro for LV [35] and two Belgian strains [36]. This region and the corresponding region in GP3 were also shown to be immunogenic for other isolates, including a Danish deletion mutant with an 8 aa deletion [34, 37]. Therefore, it is very likely that the highly variable region in the overlap of GP3 and GP4 is under negative selection pressure, causing deletions or mutations to occur [38]. This is in line with the existence of very similar aa sequences next to the deletion in the Austrian isolates and their nearest relatives GER09-613 and KNU-07, indicating that the four strains evolved in a similar direction, probably due to immunological pressure. The 12 aa deletion in AUT14-440 might prevent the binding of GP4-specific antibodies and thereby result in an evolutionary advantage. This idea will be tested in further experiments.

Another interesting aspect of the deletion is the fact that the heterotrimeric minor glycoproteins GP2, GP3 and GP4 are abundant on the viral surface and probably determine cell tropism [39]. A deletion of 12 aa in two of these proteins might lead to an altered cell tropism, as seen in this study for AUT14-440, which is, despite its type 1 genotype, capable of infecting MARC-145 cells. The single nt deletion in the 3′UTR of AUT14-440 has not been described in other PRRSV-1 strains, although there is a report of a deletion at a different position within the 3′UTR in highly pathogenic Chinese strains [40]. As the 3′UTR is essential for PRRSV replication [41], deletions in this area might have an implication for the replication efficacy of the virus.

AUT14-440 differed from the other Austrian isolate AUT13-883 and the German isolate GER09-613 also in other aspects. It was the only strain to cause clear respiratory distress in pigs in the animal trial. Animals infected with AUT14-440 ceased to increase daily weight gain in the second week after infection, highlighting the economic relevance of the strain. Although pigs from the other groups remained relatively healthy throughout the trial, moderate to severe lung lesions were found in pigs infected with AUT13-883, which also showed varying developments of daily weight gain. It is conceivable that clinical signs would have been more prominent in all infection groups if a less natural route of infection, e.g., intramuscular, or higher viral titres had been chosen.

Viremia was already detected 3 dpi in pigs infected with the Austrian strains, which is in accordance with other experimental infections [42, 43], whereas the GER09-613 group showed the first viral RNA and titres in serum 10 dpi. A likely explanation for the late onset and the lack of clinical signs is that strain GER09-613 was cell-culture adapted on MARC-145 cells and hence potentially attenuated and thus had to re-adapt to the host. Future studies will be necessary to address the sequence differences between inoculated GER09-613 and the re-isolated virus.

In the present study, high amounts of viral RNA in blood were associated with severity of clinical signs, with titres of AUT14-440 exceeding those of the other strains by up to 100-fold. In contrast, the amounts of viral RNA in nasal swabs were lowest for pigs infected with AUT14-440, indicating that the amount of virus shedding via the respiratory route is not necessarily correlated with viremia. The detection of virus shedding 3 dpi is probably an artefact of intranasal infection, as pigs from group GER09-613 do not show any viral RNA in nasal swabs 7 dpi, which corresponds to the late onset of viremia in this group.

The early detection of non-neutralizing N-specific antibodies is in accordance with previous studies [42, 43]. The IDEXX X3 ELISA is not quantitative but the high P/PK ratios of group AUT14-440 are in agreement with the high number of viral genome copies in the blood of animals infected with this strain.

In summary, we have determined the complete genome sequence of two recent Austrian field isolates of PRRSV and interpret the nucleic acid sequences and the phylogeny in the light of the strains’ in vivo characteristics. Both isolates cluster with a German field isolate from 2009 and the South Korean KNU-07 strain in whole genome phylogenetic analysis. One of the field isolates, AUT14-440, caused clinical signs in an animal experiment, grew on MARC-145 cells and showed exceptional deletions. These well characterized isolates represent an excellent basis for further studies on the implications of the described molecular properties on virus entry, replication and pathogenicity. It remains unclear whether the similarities to East Asian strains are unique to European strains because of the lack of full genome sequences of strains currently present in the field. As PRRSV strains are subject to constant evolutionary pressure, both from vaccination and from instruments of modern herd management, it is of the utmost importance to keep up with the increasing diversity of PRRSV by studying current isolates.

## Additional files

10.1186/s13567-015-0293-x **Phylogenetic analysis of ORF5.** Phylogenetic tree based on ORF5 nucleotide sequences of 41 PRRSV-1 strains and PRRSV-2 prototype VR2332 as an out-group as well as the two closest related sequences in NCBI BLASTn phylogenetic tree for each isolate. The PRRSV strains presented in this study are marked in red. The tree was constructed using the neighbour joining method with the numbers at the nodes representing bootstrap values in % of 1000 replicates. Scale bar: number of substitutions per site.
10.1186/s13567-015-0293-x **Phylogenetic analysis of ORF7.** Phylogenetic tree based on ORF7 nucleotide sequences of 41 PRRSV-1 strains and PRRSV-2 prototype VR2332 as an out-group as well as the two closest related sequences in NCBI BLASTn phylogenetic tree for each isolate. The PRRSV strains presented in this study are marked in red. The tree was constructed using the neighbour joining method with the numbers at the nodes representing bootstrap values in % of 1000 replicates. Scale bar: number of substitutions per site
