# Supplementary figures and images for: Characterization of two Austrian porcine reproductive and respiratory syndrome virus (PRRSV) field isolates reveals relationship to East Asian strains
Source: Vet Res. 2016 Jan 11;47:17. doi: 10.1186/s13567-015-0293-x (PMC4709888; doi:10.1186/s13567-015-0293-x)

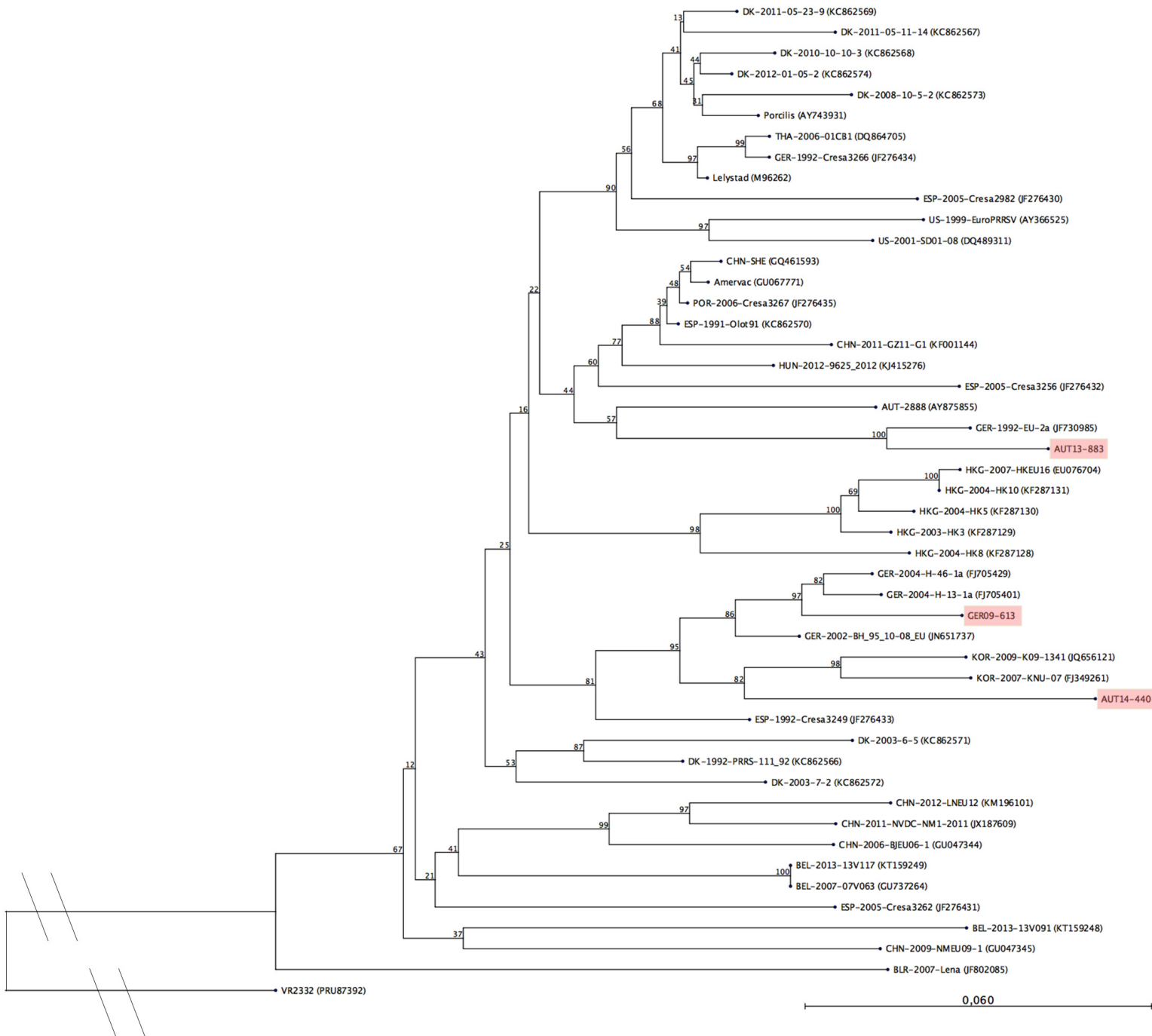

Supplement: Supplementary file 1 — 10.1186/s13567-015-0293-x Phylogenetic analysis of ORF5. Phylogenetic tree based on ORF5 nucleotide sequences of 41 PRRSV-1 strains and PRRSV-2 prototype VR2332 as an out-group as well as the two closest related sequences in NCBI BLASTn phylogenetic tree for each isolate. The PRRSV strains presented in this study are marked in red. The tree was constructed using the neighbour joining method with the numbers at the nodes representing bootstrap values in % of 1000 replicates. Scale bar: number of substitutions per site. [file 13567_2015_293_MOESM1_ESM.pdf]

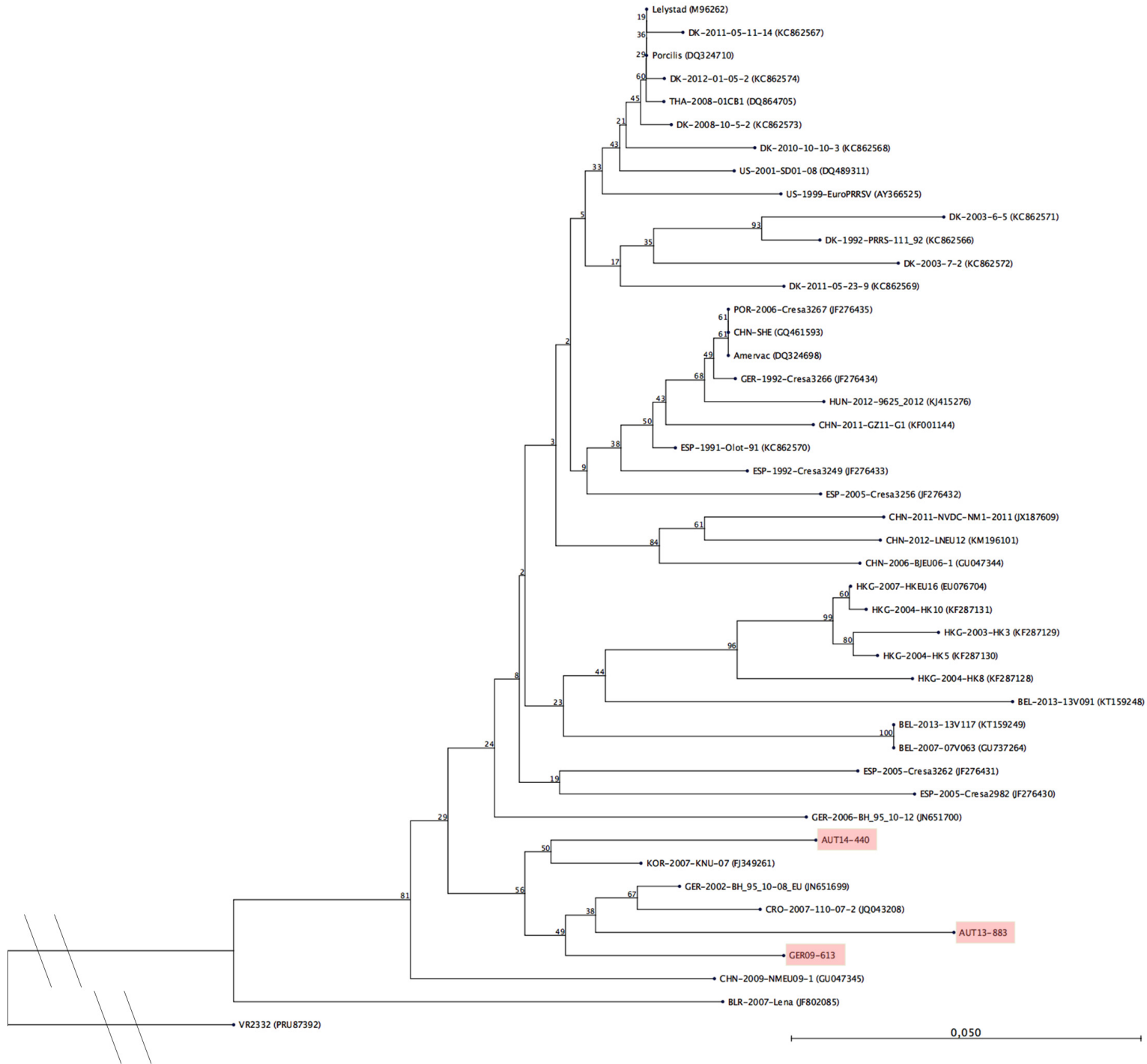

Supplement: Supplementary file 2 — 10.1186/s13567-015-0293-x Phylogenetic analysis of ORF7. Phylogenetic tree based on ORF7 nucleotide sequences of 41 PRRSV-1 strains and PRRSV-2 prototype VR2332 as an out-group as well as the two closest related sequences in NCBI BLASTn phylogenetic tree for each isolate. The PRRSV strains presented in this study are marked in red. The tree was constructed using the neighbour joining method with the numbers at the nodes representing bootstrap values in % of 1000 replicates. Scale bar: number of substitutions per site [file 13567_2015_293_MOESM2_ESM.pdf]
